# Effect of Heavy Metal Stress on Phenolic Compounds Accumulation in Winter Wheat Plants

**DOI:** 10.3390/molecules28010241

**Published:** 2022-12-28

**Authors:** Marta Jańczak-Pieniążek, Jan Cichoński, Patrycja Michalik, Grzegorz Chrzanowski

**Affiliations:** 1 Department of Crop Production, Institute of Agricultural Sciences, Land Management and Environmental Protection, University of Rzeszow, Zelwerowicza 4 St., 35-601 Rzeszow, Poland; 2 Doctoral School, University of Rzeszow, st Rejtana 16C, 35-959 Rzeszow, Poland; 3 Department of Biotechnology, Institute of Biology and Biotechnology, University of Rzeszow, Zelwerowicza 8B, 35-601 Rzeszow, Poland

**Keywords:** *Triticum aestivum* L., hybrid wheat cultivars, copper (Cu), lead (Pb), seed germination, phenylalanine ammonia-lyase (PAL), tyrosine ammonia-lyase (TAL), total phenols, flavonoids

## Abstract

Heavy metal stress can lead to many adverse effects that inhibit cellular processes at various levels of metabolism, causing a decrease in plant productivity. In response to environmental stressors, phenolic compounds fulfill significant molecular and biochemical functions in plants. Increasing the biosynthesis of phenolic compounds in plants subjected to heavy metal stress helps protect plants from oxidative stress. A pot experiment was carried out to determine the effect of the accumulation of copper (Cu) and lead (Pb) salts at concentrations of 200, 500, and 1000 ppm on seed germination, the activity of enzymes in the phenylalanine ammonia-lyase pathway (PAL) and tyrosine ammonia-lyase (TAL), along with the total phenol and flavonoid contents in seedlings of hybrid *Triticum aestivum* L. (winter wheat) cultivars. The accumulation of heavy metals, especially Cu, had a negative impact on the seed germination process. The cultivar “Hyacinth” reacted most strongly to heavy metal stress, which was confirmed by obtaining the lowest values of the germination parameters. Heavy metal stress caused an increase in the activity of PAL and TAL enzymes and an increase in the accumulation of phenolic compounds. Under the influence of Cu, the highest activity was shown in cv. “Hyvento” (especially at 200 ppm) and, due to the accumulation of Pb, in cv. “Hyacinth” (1000 ppm) and cv. “Hyking” (200 ppm). The cultivar “Hyking” had the highest content of phenolic compounds, which did not increase with the application of higher concentrations of metals. In other cultivars, the highest content of total phenols and flavonoids was usually observed at the lowest concentration (200 ppm) of the tested heavy metals, Cu and Pb.

## 1. Introduction

Heavy metals are elements that naturally occur on Earth [1]. However, industrial and agricultural activities related to the use of chemical fertilizers and pesticides, sewage sludge, and manure composts may cause their increased accumulation in the soil, which has long-term effects on the ecosystem [2,3,4]. Additionally, heavy metals may influence human health, due to the consumption of plant products from crops grown in areas contaminated with heavy metals [5,6,7].

Heavy metals include both essential and non-essential metals yielding symptoms of toxicity. Some heavy metals, such as Cu, Zn, Mn, Ni, and Fe, play a significant role in the functioning of plants as an important component of photosystems and enzymes. Therefore, these metals in low concentrations are of key importance for metabolic activity [8]. If a certain concentration threshold is exceeded, these metals cause adverse effects, which are associated with their toxic effects on plants [9]. Other metals, such as Cd, Hg, Pb, and Cr, are not essential for plants and are considered more problematic [3].

Copper (Cu) is an element that belongs to micronutrients and plays a significant role in the assimilation of CO_2_ and in the synthesis of ATP in plants. Cu is also an important component of proteins such as plastocyanin, which is part of the photosynthetic system, and cytochrome oxidase, which is part of the respiratory electron transport chain [10]. However, the excess occurrence of this element in the soil has a cytotoxic effect on plants, causing oxidative stress, which in turn leads to plant damage [11].

Lead (Pb) is a heavy metal that is not essential for the functioning of plants and does not play any role in the cell metabolism process. This element is easily absorbed and accumulated in various parts of the plant. High concentrations of Pb can cause a number of toxic symptoms in plants. These include, among others, the inhibition of growth, disturbances in the photosynthesis process, mineral nutrition and water management, and adverse effects on the structure and permeability of the cell membrane [12,13,14].

Other authors have previously described the toxic effect of heavy metals on plants [15,16,17,18]. The exposure of plants to heavy metals can lead to many negative effects that inhibit the cellular processes at various levels of metabolism [19,20,21]. Due to their sessile nature, plants are exposed to abiotic stresses, including the adverse effects of heavy metals. Therefore, they have evolved different mechanisms against these stress factors [3]. One of the early plant responses to heavy metal stress is the production of reactive oxygen species (ROS) [22], which are classified as radicals: superoxide (O_2_^•−^) and hydroxyl radicals (^•^OH), as well as non-radicals, such as hydrogen peroxide (H_2_O_2_) and singlet oxygen (^1^O_2_). ROS production in plants is located in chloroplasts, mitochondria, and peroxisomes [23,24]. The action of ROS can cause damage at physiological and biochemical levels, resulting in decreased cell membrane stability, photosynthesis efficiency, compromised pigment production, hormonal and nutrient imbalances, and the inhibition of DNA replication, gene expression, and cell division [3,25]. In plant tissues, ROS can be produced directly by Cu, Pb, Cr, and As and indirectly by Cd, because it is redox-inactive and can produce ROS by inactivating enzymes [26].

In the event of an imbalance between the production and degradation of ROS, plants have developed an antioxidant system that consists of enzymatic antioxidants, which include superoxide dismutase, catalase, peroxidase, ascorbate peroxidase, guaiacol peroxidase, glutathione reductase, monodehydroascorbate reductase, and dehydroascorbate reductase [20,23]. Plant cells also produce low molecular non-enzymatic antioxidants, which are involved in ROS removal. Depending on the chemical structure, phenolic compounds are divided into several groups, simple phenols, benzoic acids, phenylpropanoids, and flavonoids [3,27,28,29,30]. Phenolic compounds are, therefore, classified as beneficial antioxidants that can scavenge ROS in plants exposed to stress factors [31].

Phenolic compounds are mainly created through the synthesis of cinnamic acid, which is formed from aromatic l-amino acids as a result of the action of phenylalanine ammonia-lyase (PAL) (EC 4.3.1.24), which causes phenylalanine deamination to form *trans*-cinnamic acid. The next step is the hydroxylation of this acid by cinnamate 4-hydroxylase (C4H, EC 1.14.13.11) and the production of *p*-coumaric acid, which can also be produced by tyrosine ammonia-lyase (TAL, EC 4.3.1.23), bypassing the C4H reaction [32,33]. Cinnamic and coumaric acids are then used as intermediate substrates for further phenylpropanoid acids and flavonoids [34].

Phenolic compounds are signaling molecules in the defense mechanism of plants, and they mediate the transport of auxin. In addition, some phenolic compounds can mitigate the toxic effects of heavy metals by increasing plant tolerance [17,35]. Therefore, the concentration of phenols in plant tissues is a good indicator that allows researchers to predict the range of tolerance to the stress factors that occur in plants [29]. Increasing the biosynthesis of phenolic compounds in plants subjected to heavy metal stress helps protect plants from oxidative stress. Furthermore, flavonoids enhance the process of metal chelation, which may reduce the level of harmful hydroxyl radicals in plant cells [36]. Flavonoids play a protective function in stress conditions by neutralizing radicals before they damage cells. They are included among the essential secondary metabolites synthesized in almost all parts of plants as part of plant–environment communication [37].

Wheat (*Triticum aestivum* L.) is a strategic and important crop due to its high yield, chemical composition, and technological properties of the grain [38]. Currently, the world cultivation area is 219 million hectares, while the annual production is 761 million tonnes of grain [39]. The yield and quality of wheat grain depend on the genotype, environmental factors, and the interactions between them [40,41]. Abiotic stress is classified as an environmental factor that affects wheat yield. As a result of stress factors, the growth of wheat plants may be disturbed, causing a decrease in the level of yield. One of the strategies to improve yield stability under various environmental conditions is to use the vigor of hybrid cultivars. Therefore, there is an increasing interest in the cultivation and use of hybrid wheat [42,43]. The increase in the yield level due to the cultivation of hybrid cultivars can range from 5 to 20%, which results from the improvement of yield stability under biotic and abiotic stress conditions, compared to traditional pure-line cultivars [44,45,46,47,48]. However, the use of hybrid wheat cultivars by farmers is not common, which is caused mainly by the high cost of seed production (due to the low seed set on male-sterilized female lines) [49].

There is a lack of information in the literature on the impact of heavy metals on the antioxidant system of hybrid wheat cultivars, in particular on the content of phenolic compounds. This study aimed to demonstrate the response of hybrid wheat cultivar seedlings to stress caused by heavy metals (Cu and Pb) in the soil. It is assumed that heavy metals, applied in various concentrations, will cause a different reaction in wheat plant cultivars to oxidative stress. This study examined the role of phenolic compounds under the conditions of heavy metal-induced oxidative stress. The seed germination and activity of enzymes involved in the biosynthesis of phenolic compounds (PAL and TAL) were also evaluated.

## 2. Results

### 2.1. Germination Test

The accumulation of heavy metals, especially Cu, had a negative effect on the germination of wheat seeds (Table 1). Compared to the control, a decrease in germination rate and germination potential was observed in most cases. Only in the case of Pb application at a concentration of 200 ppm was it recorded that no significant differences were found in the values of germination potential (the cultivar “Hyking”), and germination rate and potential (the cultivar “Hyvento”). A decrease in the germination parameters was observed with the increases in both Cu and Pb concentrations. Increasing the Cu concentration did not significantly differentiate the germination rate and germination potential values, except for the lowest concentration (200 ppm) in the cultivar (cv.) “Hyvento”, which increased the germination potential values compared to the other concentrations. In the case of Pb, the used concentrations significantly differentiated the value of the germination parameters. Each cultivar showed a decrease in its values as a result of the 1000 ppm concentration, compared to the 200 ppm concentration. Among the hybrid wheat cultivars tested, cv. “Hyacinth” was the most susceptible to the presence of lead, which was observed, in particular, when a concentration of 1000 ppm was used, resulting in the lowest germination rate and germination potential parameters.

### 2.2. Activity of the Phenylpropanoid Pathway Enzymes

**Figure 1:**
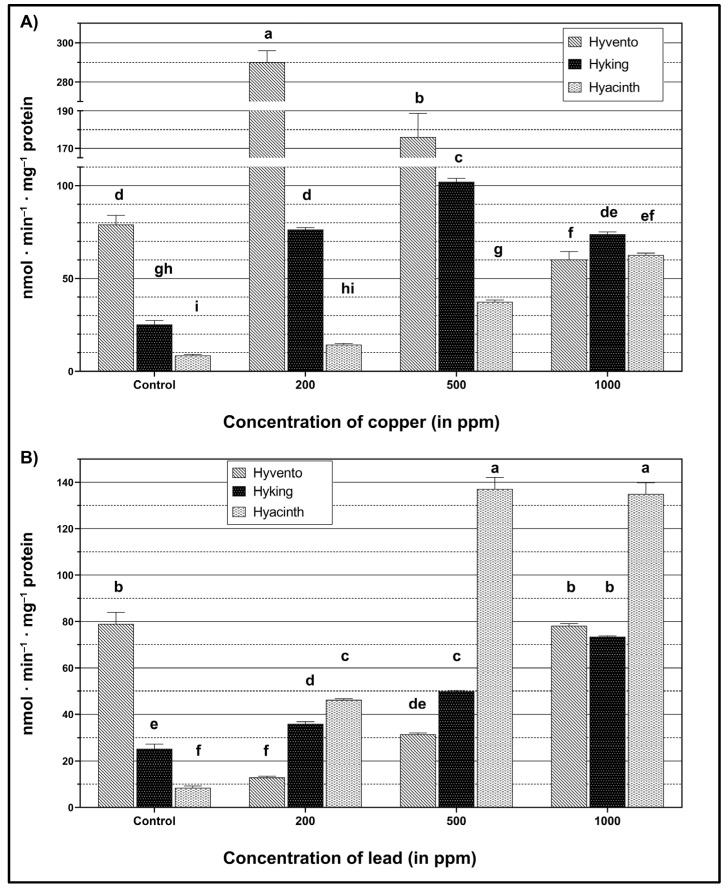
Activity of l-phenylalanine ammonia-lyase within wheat cultivars under heavy metal treatment. (**A**) The effect of copper (Cu), F_2,24_ = 2188.96, *p* < 0.0001 (cultivar); F_3,24_ = 690.17, *p* < 0.0001 (concentration); F_6,24_ = 575.35, *p* < 0.0001 (cultivar × concentration). (**B**) The effect of lead (Pb), F_2,24_ = 663.28, *p* < 0.0001 (cultivar); F_3,24_ = 1202.14, *p* < 0.0001 (concentration); F_6,24_ = 660.68, *p* < 0.0001 (cultivar × concentration). The bars for each metal marked by different letters are statistically different at *p* < 0.05 (Tukey’s test).

**Figure 2:**
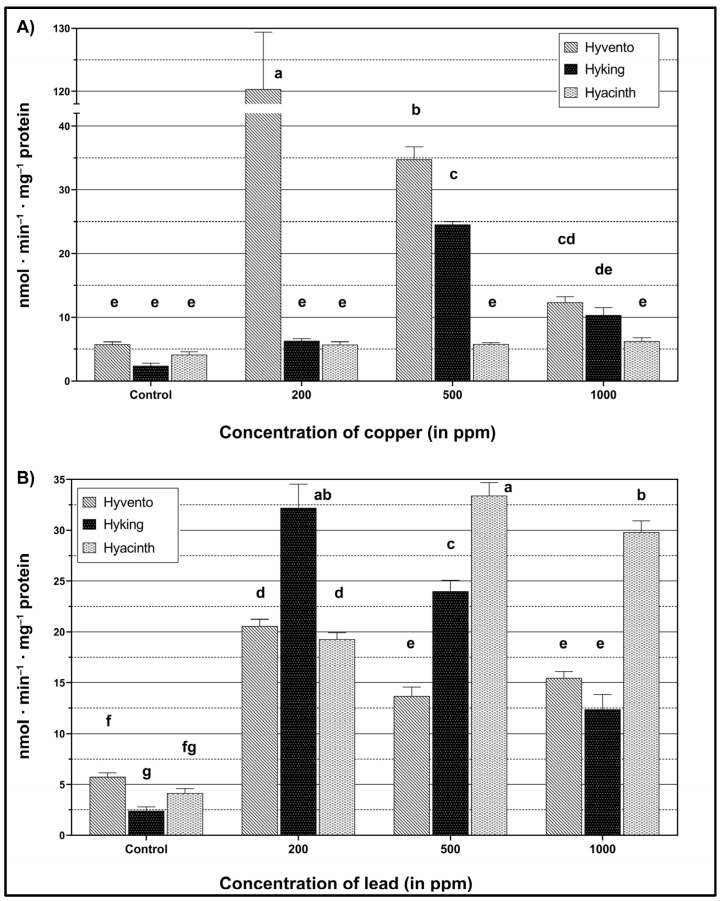
Activity of l-tyrosine ammonia-lyase within wheat cultivars under heavy metal treatment. (**A**) The effect of copper (Cu), F_2,24_ = 163.18, *p* < 0.0001 (cultivar); F_3,24_ = 92.15, *p* < 0.0001 (concentration); F_6,24_ = 94.66, *p* < 0.0001 (cultivar × concentration). (**B**) The effect of lead (Pb), F_2,24_ = 151.75, *p* < 0.0001 (cultivar); F_3,24_ = 659.12, *p* < 0.0001 (concentration); F_6,24_ = 147.52, *p* < 0.0001 (cultivar × concentration). The bars for each metal marked by different letters are statistically different at *p* < 0.05 (Tukey’s test).

The experiment has shown the influence of heavy metals on the activity of the phenylpropanoid pathway enzymes, PAL and TAL, in wheat seedlings of the tested hybrid wheat cultivars (see Figure 1 and Figure 2). Significantly higher activity of the PAL enzyme was found, compared to that of TAL.

#### 2.2.1. PAL Activity

In all tested cultivars (except for cv. “Hyacinth”), the greatest increase in PAL activity, compared to the control, was the result of using the lowest (200 ppm) Cu concentration (Figure 1A). Compared to the other cultivars, cv. “Hyvento” showed the highest PAL activity at a 200 ppm concentration (from cvs. “Hyking” and “Hyacinth”, with 213.73 and 275.67 mol min^−1^ mg^−1^ protein, respectively). The use of higher concentrations of Cu (500 and 1000 ppm) in cv. “Hyvento” resulted in a decrease in PAL activity by 267.1 and 122.8%, respectively, compared to the control. The “Hyking” cultivar showed an increase in PAL activity (200 and 500 ppm) proportional to the concentrations used, along with a decrease as a result of the 1000 ppm concentration application. A different situation was found in cv. “Hyacinth”, in which PAL activity increased proportionally to the concentrations of Cu used.

PAL activity increased with rising Pb concentrations (Figure 1B), relative to the control. The exception was the “Hyvento” cultivar, which showed a significant decrease in PAL activity as a result of Pb application. In particular, the application of the 200 ppm concentration resulted in a decrease in PAL activity by as much as 511.1%. With the use of higher concentrations of Pb, the activity of the enzyme increased to the control level at a concentration of 1000 ppm. The “Hyacinth” cultivar showed the highest increase in activity of this enzyme under the influence of the 500 and 1000 ppm concentrations (the values were not significantly different from each other).

#### 2.2.2. TAL Activity

Both the wheat cultivars and the metal concentrations used influenced the differentiation of TAL activity (Figure 2). No effect of Cu on TAL activity was observed in the cv. Hyacinth (Figure 2A). After applying the 200 ppm concentration, only the cv. Hyvento showed a significant increase (as much as 257.1%) in the TAL activity in relation to the control, while in the case of the other cultivars no significant effect of this concentration was demonstrated. Higher concentrations of 500 and 1000 ppm in the cv. Hyvento caused a decrease in TAL activity, which reached the control level when 1000 ppm was applied. A different situation was found in the case of the “Hyking” cultivar, where the increase in TAL activity in relation to the control occurred only as a result of exposure to 500 ppm concentration.

In the case of Pb accumulation, all the tested cultivars showed a significant increase in TAL activity compared to the control (Figure 2B). In the “Hyvento” and “Hyking” cultivars, the highest increase in TAL activity was observed at the 200 ppm concentration, while in the case of higher concentrations (500 and 1000 ppm), a decrease in TAL activity was observed. In the “Hyacinth” cultivar, Pb application led to an increase in TAL activity. The highest increase in TAL activity was observed after the application of 500 ppm Pb and was higher than the concentrations of 200 and 1000 ppm, by 73.2% and 12.1%, respectively.

### 2.3. Total Phenols

**Figure 3:**
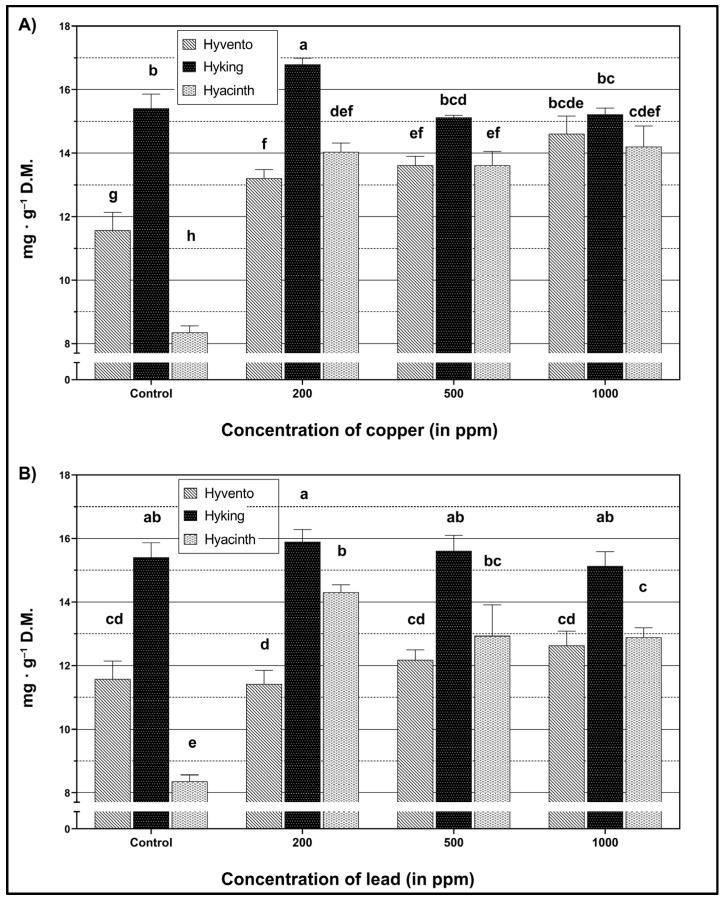
Content of total phenols within wheat cultivars under heavy metal treatment. (**A**) The effect of copper (Cu), F_2,24_ = 210.65, *p* < 0.0001 (cultivar); F_3,24_ = 115.44, *p* < 0.0001 (concentration); F_6,24_ = 44.07, *p* < 0.0001 (cultivar × concentration). (**B**) The effect of lead (Pb), F_2,24_ = 212.84, *p* < 0.0001 (cultivar); F_3,24_ = 36.05, *p* < 0.0001 (concentration); F_6,24_ = 29.13, *p* < 0.0001 (cultivar × concentration). The bars for each metal marked by different letters are statistically different at *p* < 0.05 (Tukey’s test).

The highest content of total phenols was noted in the “Hyking” cultivar (Figure 3).

In all tested wheat cultivars, the content of total phenols increased as a result of the applied concentrations of Cu (Figure 3A). The application of Cu led to an increase in the content of total phenols in cv. “Hyvento” only, along with rising Cu concentrations. Conversely, in the “Hyacinth” cultivar, Cu application caused a significant increase in the value of the tested parameter in relation to the control, which remained at a similar level in all tested concentrations. The application of 1000 ppm concentrations did not significantly differentiate the total phenol contents of each cultivar.

As a result of Pb accumulation, changes in the total phenols were found only in the “Hyacinth” cultivar (Figure 3B). The largest increase in the value of this parameter (by 71.2%) was observed as a result of the application of the lowest concentration of lead (200 ppm), compared to the control. Under the influence of higher concentrations, the content of total phenols decreased, particularly due to the application of 1000 ppm concentrations. In the “Hyvento” and “Hyking” cultivars, there were no significant changes in total phenols associated with increasing Pb concentrations.

### 2.4. Flavonoids

**Figure 4:**
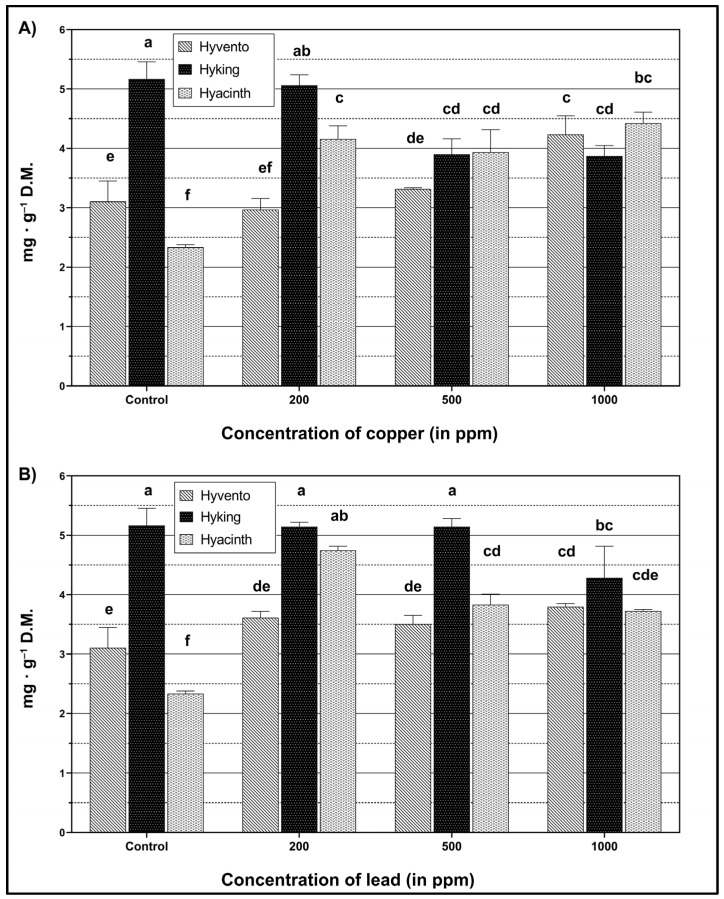
Content of flavonoids within wheat cultivars under heavy metal treatment. (**A**) The effect of copper (Cu), F_2,24_ = 66.10, *p* < 0.0001 (cultivar); F_3,24_ = 13.78, *p* < 0.0001 (concentration); F_6,24_ = 37.53, *p* < 0.0001 (cultivar × concentration). (**B**) The effect of lead (Pb), F_2,24_ = 152.16, *p* < 0.0001 (cultivar); F_3,24_ = 30.34, *p* < 0.0001 (concentration); F_6,24_ = 23.75, *p* < 0.0001 (cultivar × concentration). The bars for each metal marked by the different letters are statistically different at *p* < 0.05 (Tukey’s test).

An increase in flavonoid content as a result of Cu stress in the “Hyvento” cultivar was observed only in the iteration at 1000 ppm concentration (Figure 4A). In relation to the control, the increase was 6.7%. In the “Hyking” cultivar, a gradual decrease in the content of flavonoids was found with increasing Cu concentration. However, there were no statistically significant differences in the contents of flavonoids between the control and the concentration of 200 ppm, and between the concentrations of 500 and 1000 ppm. In the case of the “Hyacinth” cultivar as a result of the application of the 200 ppm concentration, the content of flavonoids was significantly increased by 78.1% compared to the control. The use of 500 and 1000 ppm concentrations did not cause significant changes in the value of the tested parameter.

The application of Pb resulted in a substantial increase in the content of flavonoids in relation to the control, only in the case of the “Hyacinth” cultivar (Figure 4B). As a result of the application of the concentration at 200 ppm, an increase of as much as 103.1% was found, but higher concentrations (500 and 1000 ppm) resulted in a decrease in flavonoid levels. In the “Hyking” cultivar, which was characterized by the highest content of flavonoids compared to the other cultivars, there was a significant change in the content of flavonoids as a result of the 1000 ppm Pb application, which resulted in a decrease of 20.6% compared to the control and 20.1% compared to the concentrations 200 and 500 ppm.

## 3. Discussion

Heavy metal pollution is a major problem for plants, resulting in a disruption of plant metabolism caused by a series of interactions occurring at the cellular level. The toxic effects of heavy metals can result from their binding to sulfhydryl groups in proteins, leading to the inhibition of function and damage to the protein structure [20,50]. Such a problem, related to the accumulation of heavy metals, is common in Poland and European Union countries in cultivated areas. These pollutants come mainly from the use of intensive fertilization programs in agricultural field areas and their location near roads and industrial factories. The indicators of soil contamination with heavy metals, including Cu and Pb, are given in the works of Dach and Starmants [51] and Alengebawy et al. [15]. One example of a defense mechanism against the negative effects of heavy metals is the biosynthesis of phenolic compounds, which are involved in plant responses to environmental stresses [31,52].

The present study investigated the effects of two heavy metals, Cu and Pb, on seed germination, PAL and TAL enzyme activities, total phenols, and the flavonoid content of selected hybrid winter wheat cultivars (“Hyvento”, “Hyking”, and “Hyacinth”).

As a result of the accumulation of heavy metals, seed germination, which is one of the most important processes that occur in the plant life cycle, is disturbed [53]. Hydrolytic enzymes, such as acid phosphatases, proteases, and α-phosphatase play a significant role in seedling growth and development by mobilizing nutrients in the endosperm. In the presence of heavy metals, starch is immobilized, and the source of nutrients is reduced due to a decrease in the activity of proteolytic enzymes [4]. The reduction of seed germination, caused by heavy metal stress, can also result from disturbances in cell membrane permeability and osmotic balance [54].

This was also demonstrated in our research, in which the application of solutions of heavy metal salts (Cu and Pb) negatively affected the process of seed germination. With the increase in the concentrations of metals, a decrease in the value of the germination parameters was observed. The accumulation of Cu caused a very strong decrease in the germination rate and germination potential, but no significant differences were found between the various concentrations of this metal. The studies also showed that Cu, compared to Pb, inhibited seed germination to a much higher extent, which was reflected in the germination rate and germination potential obtained.

Previous studies by other authors have shown that the accumulation of Pb in the soil, even at very low concentrations, inhibits seed germination and slows the growth of seedlings, which is confirmed by reduced values of germination parameters [12,13]. Lamhamdi et al. [55], when examining wheat seedlings, demonstrated that the presence of Pb induces large disturbances in uptake by plants, causing adverse metabolic changes leading to the inhibition of seedling growth. The accumulation of Pb in plants increases with increasing exogenous levels in the substrate, which can lead to physiological and biochemical dysfunction, negatively affecting seed germination [56]. The higher toxicity of Cu during the germination of wheat seeds may occur due to the stronger influence of this metal on amylase and protease activities, causing the inhibition of nutritional supply to the developing seedlings [57]. This fact can be explained by the reduction in the germination rate that is associated with Cu accumulation, due to the inhibition of starch and sucrose decomposition in reserve tissue by a decrease in the activity of alpha-amylase and invertase isoenzymes [58].

In response to environmental stress, plants are capable of activating secondary metabolic pathways, as is exemplified by the metabolism of phenylpropanoids [59]. This pathway produces a wide variety of secondary phenolic metabolites and lignins, which are one of the main components of the plant cell wall [60]. Activation of the phenylpropanoid pathway is a source of non-enzymatic antioxidants that protect plants from oxidative stress, caused by the presence of heavy metals [61]. Phenylalanine ammonia-lyase (PAL) and tyrosine ammonia-lyase (TAL) are the key enzymes involved in this pathway [62].

In our research, the activity of PAL and TAL enzymes was differentiated by the concentration of heavy metals and wheat cultivars. In most cases, the accumulation of both Pb and Cu resulted in an increase in the activity of these enzymes at a concentration of 200 ppm. Greater enzyme activity in the phenylpropanoid pathway was also found as a result of Cu application, compared to Pb.

An increase in PAL and TAL activity was also shown by other authors in response to the occurrence of both biotic and abiotic environmental stresses [60,63,64,65,66].

In our research, much higher activity of the PAL enzyme was shown compared to TAL. This is also confirmed by the results of an experiment carried out on *Scrophularia striata* by Beshamgan et al. [66], which showed a two-fold higher increase in PAL activity compared to TAL due to cadmium accumulation. A higher contribution of PAL in relation to TAL in flavonoid biosynthesis was also found by Feduraev et al. [60] in studies on wheat seedlings and by Khan et al. [67] after the application of the elicitors chitin and chitosan to soybean leaf tissues. This can be explained by the different functions of PAL and TAL, which can be differently regulated by specific lignin pathway intermediates, and via organized multienzyme complexes [68].

In this work, the impact of heavy metals on the content of phenolic compounds was also examined. The accumulation of these compounds results from the biosynthesis of phenylpropanoid enzymes, including phenylalanine ammonia-lyase, chalcone synthase, shikimate dehydrogenase, cinnamyl alcohol dehydrogenase, and polyphenol oxidase, which are dependent on metal-induced transcript-level modulation of the genes encoding biosynthetic enzymes [29]. An increased level of biosynthesis of the phenolic compounds, including flavonoids, was observed in plants exposed to biotic and abiotic stresses, which confirms the higher tolerance of plants to these stressors. Flavonoid biosynthesis pathways provide compounds involved in defense responses to a wide spectrum of biotic and abiotic factors [69]. Flavonoids form stable complexes with heavy metal ions, thus preventing the development of oxidative stress [69,70,71]. The accumulation of flavonoids in the vacuoles reduces the osmotic potential in the cells, increasing the absorption of water by plants, and thereby improving their resistance to unfavorable climatic conditions. Flavonoids also protect cells from damage caused by oxidative stress, thus preventing lipid oxidation by scavenging free radicals [69]. In our research, an increase in the content of total phenols and flavonoids was also observed due to the accumulation of heavy metals. In most cases, the highest increase in the level of total phenols and flavonoids was recorded as a result of applying the lowest concentration of metals (200 ppm). An improvement in phenylpropanoid metabolism and an increase in the level of phenolic metabolites were also found in common wheat under various environmental stresses [72]. The concentration of heavy metals is a key factor that influences the physiological response of plants and secondary metabolism. Research conducted by Ibrahim et al. [73] on the effect of various concentrations of heavy metals on plant growth proves that their lower concentration stimulates the production of secondary metabolites, which is manifested by an increase in their amount, while higher concentrations of these metals inhibit the synthesis of secondary metabolites in plants. Furthermore, as reported by González-Mendoza et al. [61], a high concentration of heavy metals causes a decrease in the accumulation of phenolic compounds, caused by the plant’s inability to synthesize new phenolic compounds and flavonoids.

The metabolism of phenols is metal-specific, influenced by the differences in the physical properties of metals [52], which was also confirmed in our research by showing the different effects of the tested metals on the accumulation of phenolic compounds. In our experiment, a different relationship was found between the activity of the PAL and TAL enzymes and the accumulation of phenolic compounds. These differences may suggest a different involvement of the phenylpropanoid pathway in the synthesis of various secondary metabolites other than phenolic compounds [61]. Therefore, it seems necessary to conduct future studies to explain the role of this pathway in the synthesis of other secondary metabolites.

In the experiment, the plants of hybrid wheat cultivars reacted differently to the abiotic stress caused by the application of heavy metals, which was confirmed by the values of the tested parameters. This may occur due to differences in the response of these cultivars, caused by their genotype, which contributes to a different response to the stress caused by heavy metals. This can be explained by the difference in the expression of resistance genes in particular cultivars of crop plants. The success of adaptation and survival under the stress conditions caused by heavy metals depends not only on the intensity of environmental factors but also on the genetic background associated with them [74,75]. Knowledge of these processes could contribute to the development of breeding strategies for sustainable crop plants, particularly hybrid cultivars.

## 4. Materials and Methods

### 4.1. Plant Material

#### 4.1.1. Germination Experiment

A germination test was carried out to select 3 cultivars of hybrid wheat for further research: “Hyvento”, “Hyking”, and “Hyacinth” (the breeder was Saaten-Union GmbH, Estrées Saint-Denis, France). Wheat seeds (50 pcs.) were placed on filter paper in 90-millimeter diameter Petri dishes saturated with 10 cm^3^ heavy metal salt solutions Cu(NO_3_)_2_ and Pb(NO_3_)_2_, at concentrations of 200, 500, and 1000 ppm. The experiment was performed in three replicates. Heavy metal concentrations were determined according to the Regulations of the Polish Minister of the Environment on the Soil Quality Standards from 5 September 2016 [16]. The control sample used distilled water instead of heavy metal salt solutions. The Petri dishes were kept at 20 °C in the dark and 65% relative humidity for 7 days.

The germination rate and potential were determined by counting the germinated seeds on the 3rd and 7th days, respectively.

Germination potential (%) = 3-day number of germinated seeds/total seed number for testing × 100%

Germination rate (%) = 7-day number of germinated seeds/total seed number for testing × 100% [76].

#### 4.1.2. Pot Experiment

A soil of clay-sand grain size (150 g) [77] was placed in plastic pots (diameter 11.5 cm, height 9.5 cm, volume 0.75 L). The soil revealed an acidic reaction (KCl; pH 6.35; H_2_O 6.52). Then, 70 cm^3^ of heavy metal salt solutions were added to each pot, and 150 wheat seeds were sown. The soil in the pots was kept at a constant moisture content of approximately 50% of the maximum water-holding capacity (WHC). After seed germination was observed, the pots were transferred to a growth chamber (model GC-300/1000, JEIO Tech Co., Ltd., Seoul, Republic of Korea), photoperiod 16 L/8 D h, temperature 22 L/20 D °C, humidity 65% RH and light intensity 350 µmol m^−2^ s^−1^. After the plants reached the appropriate stage of 2–3 leaves (12–13 BBCH scale) [78], the above-ground part of the plants was cut, treated with liquid nitrogen, and crushed. The frozen samples were then lyophilized (48 h). The lyophilized plant material was used for further research.

### 4.2. Phenolic Compounds Analysis

#### 4.2.1. Extraction of Phenolic Compounds

First, 0.2 g of plant material was extracted with 15 mL of 80% MeOH for 30 min using an ultrasonic bath (Sonic-6D, Polsonic, Warsaw, Poland). The extract was then centrifuged at 5000× *g* for 30 min. The supernatant was collected and evaporated under a vacuum at 40 °C (Hei-VAP Precision, Heidolph Instruments GmbH & Co. KG, Schwabach, Germany). The residue was suspended in water and applied onto an SPE (solid phase extraction) column, equilibrated with water. SPE was carried out with a Visiprep™ SPE Vacuum Manifold (Sigma-Aldrich, Poznan, Poland), using a Chromabond C18ec column (Macherey-Nagel GmbH & Co. KG, Dueren, Germany). The column was washed with H_2_O and the phenolic acids were eluted with methanol.

#### 4.2.2. Total Phenols Determination

Total phenolics were determined spectrophotometrically using Folin–Ciocalteau reagent (Sigma-Aldrich, Poznan, Poland). Briefly, 75 µL of the sample was mixed with 975 µL of H_2_O, then 75 µL of Folin-Ciocalteu reagent (diluted with water 1:1) was added. After 3 min of incubation in darkness at room temperature, 125 µL of 20% Na_2_CO_3_ (Sigma-Aldrich, Poznan, Poland) was added and mixed. The absorbance of the blue complex was measured at 725 nm using an Epoch microplate spectrophotometer (Biotek Instruments Inc., Winooski, VT, USA).

#### 4.2.3. Flavonoids Determination

Total flavonoids were determined using the colorimetric method described previously by Zhishen et al. [79]. First, 250 µL of plant extract was mixed with 637 µL of distilled water, then 38 µL of 5% sodium nitrite (Sigma-Aldrich, Poznan, Poland) was added. After 6 min, 75 µL of 10% aluminum chloride (Sigma-Aldrich, Poznan, Poland) solution was added and left to stand for 5 min. Then, 250 µL of 1 M NaOH (Sigma-Aldrich, Poznan, Poland) was added. The absorbance was measured at 510 nm, using an Epoch microplate reader (Biotek Instruments Inc., Winooski, VT, USA). The flavonoid content was expressed as the catechin equivalent.

### 4.3. l-Phenylalanine (PAL) and l-Tyrosine Ammonia-Lyase (TAL) Activity

To 0.2 g of plant material, 8 mL of ice-cold TRIS-HCl buffer pH 8.8 was added. The material was homogenized for a period of 10 min, with breaks of 30 s. After homogenization, the material was centrifuged for 30 min (8000× *g*). The supernatant was collected and then used for further analysis. The reaction mixture consisted of 0.25 mL of extract, 0.5 mL of substrate (l-phenylalanine or l-tyrosine, Sigma-Aldrich, Poznan, Poland), 0.5 mL of TRIS-HCl buffer (pH 8.8), and 0.25 mL of distilled water. The reaction mixture was incubated in a heating block for 30 min at 30 °C; then, 0.5 mL of TCA was added and the mixture was centrifuged for 20 min at 6000× *g*. The absorbance of the produced *trans*-cinnamic acid and *p*-coumaric acid was measured at 290 nm (PAL) and 333 nm (TAL), respectively, using an Epoch microplate spectrophotometer (Biotek Instruments Inc., Winooski, VT, USA).

### 4.4. Statistical Analysis

All chemical analyzes were performed in triplicate. The contents of total phenols, flavonoids, and enzyme activities were expressed as the mean ± standard deviation. Data were subjected to an ANOVA, with wheat cultivar and heavy metal concentrations as fixed effects. The significance of the differences between mean values was calculated, using Tukey’s multiple comparison post hoc test, to be *p* < 0.05.

## 5. Conclusions

The accumulation of heavy metals (Cu and Pb) negatively affected the seed germination process of the hybrid wheat cultivars tested. Cu has been shown to have a much stronger effect than Pb in inhibiting seed germination. The tested heavy metals inhibited the germination process more strongly in the “Hyacinth” cultivar. The accumulation of Cu and Pb caused an increase in the activity of enzymes involved in the phenylpropanoid pathway (PAL and TAL), which participates in the synthesis of phenolic compounds. Under the influence of Cu, the highest activity was shown in the “Hyvento” cultivar (especially at concentrations of 200 ppm) and also due to Pb accumulation in cv. “Hyacinth” (at 1000 ppm) and cv. “Hyking” (200 ppm). The “Hyking” cultivar was characterized by the highest content of phenolic compounds, which did not increase with increasing metal concentrations. In the other cultivars, the highest content of total phenols and flavonoids was mostly observed at the lowest concentration (200 ppm) of the tested heavy metals. Further studies will be conducted to demonstrate the contribution of specific secondary metabolites (phenolic acids and/or flavonoid compounds) in response to heavy metal stress.

## Figures and Tables

**Table 1:** Effect of copper (Cu) and lead (Pb) treatment on seed germination.

Hybrid Wheat Cultivar	Heavy Metal Concentration	Copper (Cu)		Lead (Pb)	
		Germination Rate (%)	Germination Potential (%)	Germination Rate (%)	Germination Potential (%)
Hyvento	Control	66.00c * ± 5.29	92.00d ± 4.00	66.00d ± 5.29	92.00g ± 4.00
	200 ppm	4.00a ± 2.00	12.00c ± 2.00	56.00d ± 3.46	86.00g ± 4.00
	500 ppm	0.67a ± 1.15	2.00ab ± 0.00	28.00bc ± 3.46	56.00bcd ± 2.00
	1000 ppm	0.00a ± 0.00	0.00a ± 0.00	20.00b ± 2.00	46.00b ± 5.29
Hyking	Control	58.00bc ± 6.00	90.00d ± 6.00	58.00d ± 6.00	90.00g ± 6.00
	200 ppm	2.00a ± 2.00	8.00bc ± 0.00	36.00c ± 2.00	84.00fg ± 5.29
	500 ppm	2.00a ± 2.00	4.00ab ± 2.00	34.00c ± 4.00	64.00de ± 5.29
	1000 ppm	0.00a ± 0.00	2.00ab ± 2.00	20.00b ± 4.00	50.00bc ± 3.46
Hyacinth	Control	56.00b ± 3.46	88.00d ± 2.00	56.00d ± 3.46	88.00g ± 2.00
	200 ppm	0.67a ± 1.15	6.00abc ± 2.00	32.00c ± 3.46	74.00ef ± 4.00
	500 ppm	0.00a ± 0.00	0.67a ± 1.15	28.00bc ± 2.00	58.00cd ± 2.00
	1000 ppm	0.00a ± 0.00	0.00a ± 0.00	6.00a ± 3.46	16.00a ± 2.00
Mean		15.78 ± 26.11	25.39 ± 38.05	36.67 ± 18.17	67.00 ± 22.61
Cultivar (Cv.)		F = 4.956 *p* < 0.05	F = 4.491 *p* < 0.05	F = 30.929 *p* < 0.0001	F = 36.000 *p* < 0.0001
Concentration (Co.)		F = 1036.314 *p* < 0.0001	F = 2750.127 *p* < 0.0001	F = 228.381 *p* < 0.0001	F = 307.347 *p* < 0.0001
Cv x Co		F = 2.564 *p* < 0.05	ns	F = 9.024 *p* < 0.0001	F = 12.980 *p* < 0.0001

Data shown are the mean ± standard deviation (SD), where *n* = 3. * Different letters within a column indicate significant differences, according to Tukey’s test, at *p* < 0.05); ns—non-significant.

## Data Availability

Not applicable.

## Sample Availability

Samples are available from the corresponding author upon reasonable request.
